# The role of SeDeM for characterizing the active substance and polyvinyilpyrrolidone eliminating metastable forms in an oral lyophilizate—A preformulation study

**DOI:** 10.1371/journal.pone.0196049

**Published:** 2018-04-24

**Authors:** Paloma Flórez Borges, Encarna García-Montoya, Pilar Pérez-Lozano, Enric Jo, Montserrat Miñarro, Albert Manich, Josep Maria Suñé-Negre

**Affiliations:** 1 Pharmacy and Pharmaceutical Technology Department, Faculty of Pharmacy, University of Barcelona. Barcelona, Spain; 2 Reig Jofre Group. Sant Joan Despí, Spain; 3 IQAC-CSIC. Institute of Advanced Chemistry of Catalonia. Barcelona, Spain; Kermanshah University of Medical Sciences, ISLAMIC REPUBLIC OF IRAN

## Abstract

A preformulation study of an oral lyophilisate with cetirizine dihydrochloride (CTZ) as active ingredient, mannitol and PVP K30 as bulking agents is presented. CTZ shown a humidity content of 0.150% and a spontaneous hygroscopicity of 0.200% (both determined by SeDeM diagram), demonstrating an adequate stability behavior in solid form. A design of experiments (DoE) performed with both mannitol and PVP K30, followed by a simple factorial design (3^2^) has determined the optimum combination of excipients and CTZ, and showed that a higher proportion of PVP K30 was able to prevent metastable forms generated by mannitol.

## Introduction

Oral lyophilizates (OL) are solid pharmaceutical forms, presenting the advantages of solid and liquid forms at the same time, such as precise and unique dose, good stability, reduced package size, easy to swallow (oral route), do not present any gastrointestinal obstruction risk (therefore easy to be administered in elderly and children patients) [1]; [2]; [3]; [4]; [5]. A solution, suspension or emulsion is prepared (containing the active substance), followed by a freeze-drying process, resulting in a porous solid form that dissolves instantly when in contact with saliva or water [6]. There are several active ingredients that can be found as OL (antihistamines such as ebastine and desloratadine, antidiarrheal as loperamide and serotonin 5-HT receptor antagonist such as ondansetron) [7]. The objective of this study was to develop an OL formula of a second-generation antihistamine, cetirizine dihydrochloride (CTZ), which can be found in tablets, chewable gums, extended release tablets, capsules and syrup (liquid)[7]; [8]; [9]; [10]; [11]; [12], being candidate for an OL formulation. Therefore, a preformulation study of CTZ was performed using powder x-ray diffraction method (PXRD) to determine the rate of crystallization and differentiation of polymorphs of CTZ, followed by Nuclear Magnetic Resonance, UV-Vis spectrophotometry (UV-Vis) and Infrared spectrophotometry (IR) to identify CTZ. In addition, a SeDeM diagram was performed for the acknowledge of the rheology of CTZ [13]; [14]; [15]; [16]; [17]; [18]. A previous stability-indicating study of HPLC for the same active substance was already published [19].

Excipients are the main components of a freeze-dried product in general, by giving solid content, apparent density, resistance against mechanical damage, aesthetic claim (robust freeze-dried plug aspect). Considering freeze-dried formulas (in general), excipients can balance pH, if necessary, as buffers; guarantee isotonicity for the reconstituted solution, protect a bioactive component during the freeze-drying process, confer long term stability to the dry product. As bulking agents, excipients can generate enhance mechanical resistance by augmenting the solid content of the plug, avoiding pulverization of the plug. Bulking agents such as aspartame, gelatin and mannitol increase the total solid content, creating a firm matrix (plug) structure. For example, aspartame can be used as sweetener and bulking agent, gelatin as binder and bulking agent as well. Amongst excipients, there are those with crioprotective features (such as polyvinylpyrrolidone), which are characterized by protecting components that may be damaged by the freezing process. Excipients with a high Tg’ (glass transition temperature) allow the execution of shorter freeze-drying cycles, as they rise the maximum temperature for the ice sublimation and therefore, reducing manufacturing costs. When formulating for a OL, it must be taken into account the physicochemical characteristics of the active ingredient. For instance, those that are soluble in water (which is the case of CTZ) can produce euthectic mixtures with very low Tg’, with the formation of vitreous solids which may suffer collapse (loss of structure) during the sublimation phase of the freeze-drying cycle. One way to avoid collapse is to use mannitol as bulking agent [7].

The excipients mannitol and polyvinylpyrrolidone K30 (PVP K30) [20] were proposed as bulking agents for the development of the OL matrix. Mannitol is a polyol used as non-cariogenic sweetener and a non-hygroscopic excipient which presents a cooling effect due to its negative heat of solution providing pleasant mouthfeel and sweet taste [21]. Mannitol also helps avoiding collapse during sublimation [22]. Used as diluent for oral formulations (10–90% w/w), and as bulking agent for freeze-dried formulas (20–90% w/w), it forms rigid and elegant cake, with good dispersibility properties [23]. It can present three polymorphic states when crystalized (α, β and δ) as well as a hydrate. As a hydrate, the bound water can be released during storage, compromising the stability of the drug [23]; [24].

PVP K30 is an excipient used as binder in tablets formulas and as a solubilizer in oral and parenteral liquids, and chewable tablets and freeze-dried parenteral formulas [20]; [25]. It presents a crioprotective feature providing protection for the freeze-dried formula during freezing [26]. Increases solubility of poorly soluble drugs [27]; [28]; [29]; [30]; [31]; [32]; [33]. It was also considered as bulking agent candidate for the reason that when combined with mannitol increases the fracturability of the freeze-dried cake [34]. It may increase or decrease the glass transition temperature (Tg’), which depends on the relation between PVP, Tg’ and relative humidity [35]. However, it is not commonly used in OL formulas, which makes it interesting to acknowledge whether it can be used as bulking agent. A design of experiments (DoE) and a factorial design 3^2^ among CTZ and the excipients chosen [36] was carried out [37]. Freeze-drying viability studies (FDVT) within a simple freeze-dried cycle (-40 to 40°C) were key to acknowledge with the studied concentrations of excipients, the progressive consistency of the cake (matrix). Differential Scanning Calorimetry (DSC) was the method used for detecting metastable forms and determining Tg’. Freeze-drying microscopy (FDM) was used to determine the collapse temperature (T_CO_) of all combinations of the 3^2^ factorial design.

## Material and method

### Materials

The active substance used was cetirizine dihydrochloride (Jubilant Lifesciences Ltd, India; CTZ). The excipients used for the study SeDeM were talc (Fagron, Spain), magnesium stearate (Fagron, Spain), and colloidal silicon dioxide (Aerosil ®, Fagron, Spain). The excipients used in the factorial design were mannitol (Fagron, Spain) and polyvinylpyrrolidone Ph. Eur (Fagron, Spain). The reagents used for the preparation of phosphate buffers solutions (solubility studies) were: 37% HCl (Panreac, Barcelona), NaCl (Panreac, Barcelona), NaOH (Panreac, Barcelona) and KH_2_PO_4_ (Panreac, BCN).

### Methods

#### SeDeM

Although the main objective of the study is the development of an OL, a SeDeM diagram (for direct compression purposes) was performed for CTZ to acknowledge its rheological characteristics. The SeDeM methodology [13]; [14], is a development method for application in tablet-formulation and formulation studies. It is based in the concept of quality by design (QbD) described in ICH Q8 [36] since it evaluates critical quality attributes (CQA) that have an impact on the quality of the final product. It provides information about the suitability of active ingredients and excipients in powder for direct compression. This information indicates the degree to which the substances can be successfully compressed by means of direct compression technology. The parameters considered are the following: Bulk density (Da), Tapped density (Dc), Inter-particle porosity (Ie), Carr index (IC), Cohesion index (Icd), Hausner ratio (IH), Angle of repose (α), Powder flow (t´´), Loss on drying (%HR), Hygroscopicity (%H), Particle size (%Pf) and Homogeneity index (*Iϴ*). These parameters are determined by means of the new SeDeM diagram method, based on known equations [13]; [14]; [17]; [18], and duly validated and reproducible experimental tests, as shown in Table 1.

**Table 1:** Parameters and equations used in SeDeM methodology.

Incidence	Parameter	Symbol	Unit	Equation
*Dimension*	Bulk density	Da	g/ml	Da = P/V_a_
	Tapped density	Dc	g/ml	Dc = P/V_c_
*Compressibility*	Inter-particle porosity	Ie	-	Ie = Dc-Da/Dc x Da
	Carr index	IC	%	IC = (Dc–Da/Dc) 100
*Flowability/powder flow*	Cohesion index	IcD	N	Experimental
	Hausner ratio	IH	-	IH = Dc/Da
	Angle of repose	(α)	°	Tgα = h/r
	Powder flow	t ‘‘	s	Experimental
*Lubricity/stability*	Loss on drying	%HR	%	Experimental
	Hygroscopicity	%H	%	Experimental
*Lubricity/dosage*	Particles < 50 μm	%Pf	%	Experimental
	Homogeneity index ^b^	(Iϴ)	-	Eq (1)

^a^ Hardness (N) of the tablets obtained with the product in question, alone or blended with lubricants if highly abrasive.

^b^ Determines particle size. In accordance with the percentages of the different particle-size fractions by applying Eq (1).

^c^ Eq (1) = I*ϴ*

IƟ=Fm÷[100+(dm−dm−1)Fm−1+(dm+1.dm)Fm+1+(dm−dm−2)Fm−2+⋯+(dm−dm−n)Fm−n+(dm+n−dm)Fm+n]

Fm: percentage of particles in the majority range.

Fm-1: Percentage of particles in the range immediately below the majority range

Fm+1: Percentage of particles in the range immediately above the majority range

n: Order number of the fraction under study, within a series, with respect to the majority fraction

dm: Mean diameter of the particles in the majority fraction

dm-1: Mean diameter of the particles in the fraction of the range immediately below the majority range

dm+1: Mean diameter of the particles in the fraction of the range immediately above the majority range

Once the values have been obtained following the specific methods, the next step is to convert the numeric limits for each SeDeM Diagram parameter to radius values r, in accordance with Table 2 [13]; [14]; [15]; [16]; [17]; [18]; [38].

**Table 2:** Parameters and equations used in SeDeM methodology.

Incidence	Parameter	Limit value	Radius (r)	Factor applied to *v*
*Dimension*	Bulk density	0–1 g/ml	0–10	10 *v*
	Tapped density	0–1 g/ml	0–10	10 *v*
*Compressibility*	Inter-particle porosity	0–1.2	0–10	10*v*/1.2
	Carr index	0–50 (%)	0–10	*v*/5
	Cohesion index	0–200 (N)	0–10	*v*/20
*Flowability/powder flow*	Hausner ratio	1–3	10–0	5(3-*v*)^a^
	Angle of repose	50–0 (°)	0–10	10-(*v*/5)
	Powder flow	20–0 (s)	0–10	10-(*v*/2)
*Lubricity/stability*	Loss on drying	0–10 (%)	10–0	10-*v*
	Hygroscopicity	20–0 (%)	0–10	10-(*v*/2)
*Lubricity/dosage*	Particles < 50 μm	50–0 (%)	0–10	10-(*v*/5)
	Homogeneity index	0–2 x 10^−2^	0–10	500*v*

^a^ Same equation than (30-10v)/2,but simplified.

When all radius values are 10, the SeDeM Diagram takes the form of a regular polygon, drawn by connecting the radius values with linear segments. The results obtained from earlier parameter calculations and conversions are represented by the radius. The figure formed indicates the characteristics of the product (in this case, CTZ) and each of the parameters that determine whether the product is suitable for direct compression. In this case, the SeDeM Diagram is made of 12 parameters, which would form and irregular 12-sided polygon. To determine whether the active substance is acceptable for direct compression in numerical form, the following indexes are calculated based on the SeDeM Diagram as:

Parameter index (IP)

IP = No.p ≥5/No.Pt

No.p ≥5: Indicates the number of parameters whose value is equal to or higher than 5.

No.Pt: Indicates the total number of parameters studied.

The acceptability limit would correspond to: IP≥5

Parameter profile index (IPP)

IPP = mean r ≥ 5 of all parameters

Mean r = mean value of the parameters calculated.

The acceptability limit would correspond to IPP = mean r ≥ 5

Good compression index (IGC)

(IGC) = IPP x f

Where f is the reliability factor and is calculated as follows:

f = Polygon area/circle area

The acceptability limit will be calculated by:

IGC = IPP x f > 5.

### Solubility study of CTZ at phosphate buffer solutions at pH 1.2, 4.5, 6.8, 7.0

Procedure: 100 mg of the active ingredient is weighed in a stoppered test tube of 16 mm diameter and 160 mm high. 0.1 ml of the buffer solution is added. The mixture is stirred vigorously for 1 minute and introduced into a constant temperature system at 25 ± 0.5°C for 15 minutes. If the substance does not dissolve completely, the stirring step is repeated for another minute and the test tube placed back into the system. If the substance is completely dissolved, CTZ is defined as "very soluble" in the buffer medium. If CTZ is not dissolved, 0.9 ml of buffer is added at the same test tube, and the stirring procedure repeated. If the substance is dissolved, is described as "easily soluble". If not, 2 ml of buffer is added at the same test tube and operate again according to stirring procedure. If the substance is dissolved, it is described as "soluble". If the substance does not dissolve completely, add 7 ml at the same test tube of the dissolution media and repeat the stirring procedure. If the substance does not dissolve, it is described as “slightly soluble”.

If the substance does not dissolve completely, weigh 10 mg of the active ingredient finely pulverized into another stoppered test tube, add 10 ml of buffer solution, and operate according to stirring procedure. If the substance is dissolved, it is defined as poorly soluble.

If the substance does not dissolve completely, weigh 1 mg of finely powdered active ingredient into another test tube, adding 10 ml of buffer solution and operate according to stirring procedure. If the substance is dissolved, it is described as insoluble. The preparation of buffer phosphate solutions (pH 1.2; 4.5; 6.8 and 7.0), followed Ph Eur 8.7; [39].

### Nuclear Magnetic Resonance (NMR)

Varian Gemini-400 (100 MHz) to obtain 1 H and 13 C. The chemical shifts are expressed in parts per million (ppm) with respect to the central peak of the solvent: CDCI3 d, 7.26 (H) or DMSO-d6 d, 2.49 (H) as internal patterns.

### Infrared Spectroscopy (IR)

FTIR Perkin Elmer model 1600 equipment with according to Ph. Eur 2.2.24 general method for the determination of IR spectrum.

### Powder method of X-ray diffraction (PXRD)

Diffractometer PANalytical X'Pert PRO MPD with goniometer θ/θ of 240mm radius, configuration of convergent beam with elliptic mirror and geometry of transmission with sample holder for capillary with spinner. With Radiation Cu Kα1 (λ = 1.5418 Ǻ) with Lindemann 0.7 mm diameter glass capillary; 45 kV and—40 mA of working power; Windows in the incident axis that determine the height of the beam to 0.4 mm; Windows 0.02 Soller in the incident beam and the refracted beam in radians; detector PIXcel with active length of 3,347; Θ/2θ scanning of 2 to 60° 2θcon measurement of step of 0.026 degrees and time of measurement for 200 seconds per step.

### UV-Vis Spectrophotometry (UV-Vis)

UV-Vis spectrophotometer SPECORD 205 was used for the UV-Vis wavelength scanning of the active principle, following Ph. Eur. 8.0 method of identification A of cetirizine dihydrochloride: Dissolve 20.0 mg of CTZ in 50 mL of a 10.3 g/L solution of hydrochloric acid R and dilute to 100.0 mL with the same acid. Dilute 10.0 mL of this solution to 100.0 mL with a 10.3 g/L solution of hydrochloric acid R. Spectral range of 210–350 nm. Absorption maximum at 231 nm. Specific absorbance at the absorption maximum from 359 to 381.

### Preparation of the DoE and factorial design solutions

All components were weighed, and the solutions tested were manufactured in crystal beakers by simple mixture of components, with a magnetic stirrer (see Table 3).

**Table 3:** Factorial design 3^2^.

Solution	% Mannitol*	% PVP K30*
A	2.0	3.0
B	4.5	3.0
C	7.0	3.0
D	2.0	5.0
E	4.5	5.0
F	7.0	5.0
G	2.0	1.0
H	4.5	1.0
I	7.0	1.0

*W/v

### Established freeze-drying cycle

Freeze dryer Telstar ® L-3 (Telstar, Terrassa, Spain). Stainless steel tray as support base. A 24–28 hour freeze-drying cycle, which initiates with a freezing step of -40°C of 2 hours. Primary drying step with 95–125 μBar and a secondary drying step at 40°C. A complete cycle lasts 24–28 hours. The solutions studied are poured into crystal vials and also into polyvinylchloride (PVC) molds, using automatic pipette Multipette Plus Eppendorf (Hamburg, Germany). A vacuum packaging machine Food Saver® is used to seal the OL, followed by storage in silica gel desiccant chambers. This cycle was applied in all FDVT.

### Differential Scanning Calorimetry (DSC)

DSC 821e Mettler Toledo (Toledo, USA) equipment, using STARe SW 9.30 DSC software, with the following cycle (CSIC):

$$\begin{array}{l} 25{}^{\circ}C\;to‑80\;{}^{\circ}C\;\left(‑\;10{}^{\circ}C/min\right)\\ ‑80\;{}^{\circ}C\;1.0\;min\\ ‑80\;{}^{\circ}C\;a\;25\;{}^{\circ}C\;\left(10\;{}^{\circ}C/\;min\right) \end{array}$$

DSC determined the glass transition temperature (Tg’) of the active principle, excipients mannitol and PVP K30 and the solutions obtained among them. Samples of 2% CTZ (w/v), mannitol and PVP K30, and mixtures among them were prepared and filled in a sealed recipient without applying vacuum. All samples were frozen and then progressively heated.

### Freeze-drying microscopy (FDM)

The technique was performed at the center of excellence in freeze-drying (Sant Joan Despí, Reig Jofré Group). Olympus objective, model BX51, 10 x. Liquid Nitrogen bomb LNP94-2 (Linkam Scientific Instruments Ltd) to freeze all samples studied. A small drop of sample is put on a crystal slide, while another crystal slide is put on top of the sample. The chamber is then sealed, and the sample is frozen by liquid nitrogen. Vacuum is applied and heat to sublimate the simple, using a microscope to visualize. Dried substances present a dark aspect. Using a digital camera, it captures images every two seconds. Once T_CO_ is reached, the dryer layer is closer to the sublimation interphase generating sparkling dots indicating the loss of structure (collapse). Further elevation of the temperature results in a severe, more global loss of structure. Each sample was analyzed twice (see Table 4).

**Table 4:** FDM cycle.

Rate	temperature	holding time
10° C/min	TA a -60°C	3 min (vacuum beginning)
10° C/min	-60°C a– 40° C	0 min
5° C/min	-40°C a 20°C	0 min

## Results

### SeDeM diagram

**Fig 1:**
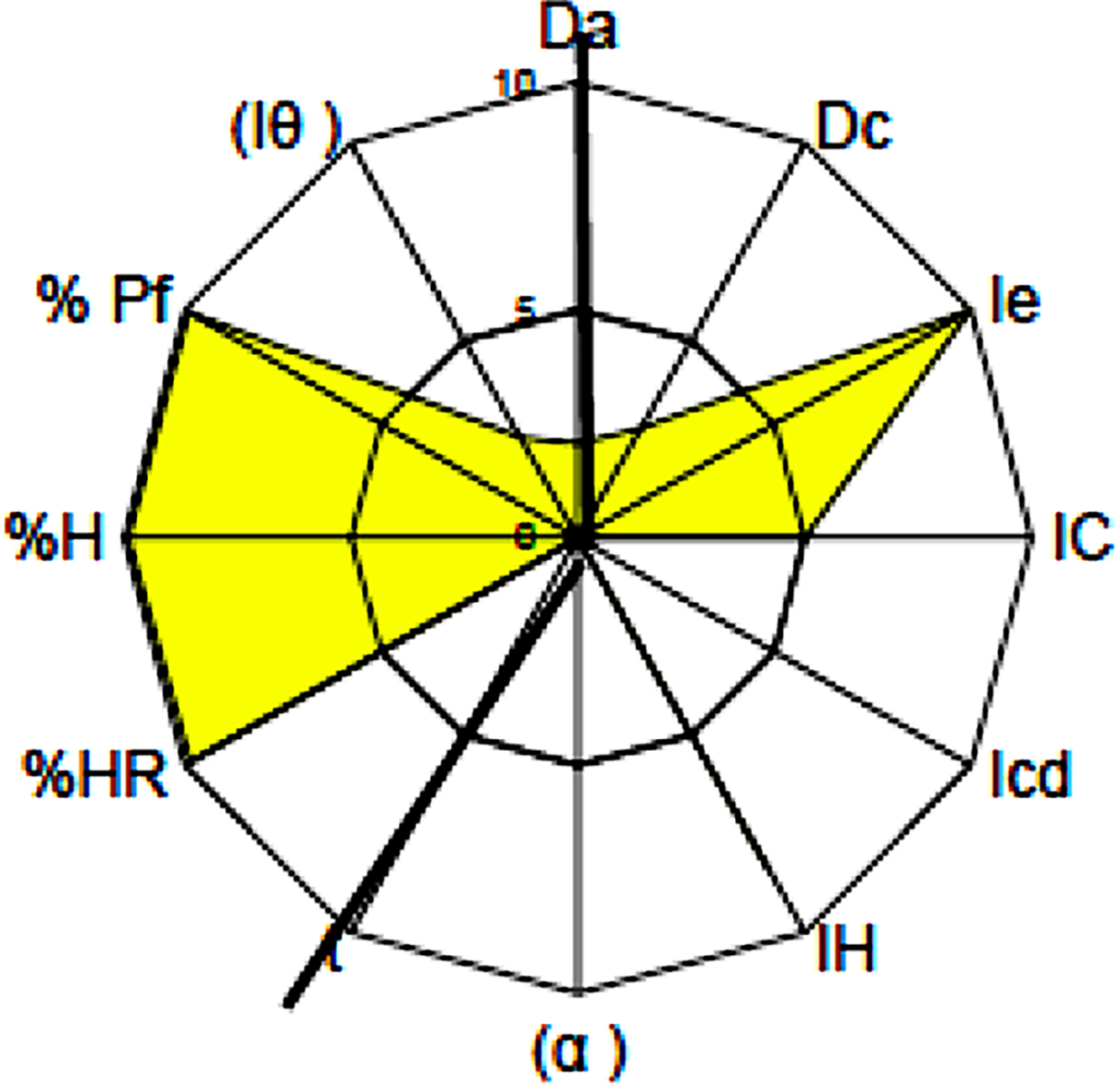
SeDeM diagram of CTZ.

CTZ presented deficient rheological parameters. The Carr index value obtained was sufficient (r = 5.00). However, the cohesion index (IcD) was null (r = 0); also, its flow parameters were also null (r = 0), such as the angle of repose and powder flow. CTZ presented particle size inferior of 0.50 μm, which is adequate for its use in a solid state (although the geometrical distribution was too broad). The parametric indexes of IP (0.42), IPP (5.03) and IGC (4.79) are insufficient for direct compression, therefore being necessary to consider the preparation of an oral lyophilisate with this active ingredient as an alternative pharmaceutical technology. The values of tapped density and bulk density (Dc = 0.276 g/ml and Da = 0.201 g/ml, respectively) indicated that CTZ presented low density and, as those values were similar, CTZ presented a good Ie (r = 0). None of the characteristics previously described has no influence in the OL manufacturing process, not interfering in the correct dispersion of CTZ (a soluble salt) in aqueous media (previous step to the freeze-drying cycle). Interestingly, from the SeDeM diagram performed, CTZ presents a humidity content of 0.150% and a spontaneous hygroscopicity of 0.200%, showing that CTZ demonstrate an adequate stability behavior in solid form hence positive for the final stability of an OL (Fig 1 and Table 5).

**Table 5:** SeDeM results of CTZ.

Incidence	Parameter	Signs	Unity	Experimental values (v)	(r)	Average incidence
Dimension	Bulk density	Da	g/ml	0.207	2.07	2.42
	Tapped density	Dc	g/ml	0.276	2.76	
Compressibility	Inter-particle porosity	Ie	-	1.208	10.00	5.00
	Carr index	IC	%	25.000	5.00	
	Cohesion Index	Icd	N	0.000	0.00	
Flowability/powder flow	Hausner ratio	IH	-	1.333	8.34	2.78
	Angle of repose	(α)	°	82.804	0.00	
	Powder flow	t	sec	Not apply	0.00	
Lubricity/stability	Loss on drying	%HR	%	0.150	9.85	9.88
	Hygroscopicity	%H	%	0.200	9.90	
Lubricity/dosage	Particles < 50 μm	%Pf	μ	0.501	9.90	6.22
	Homogeneity index	(Iθ)		0.0051	2.55	
Parameter index (IP)						0.42
Parameter profile index (IPP)						5.03
Good compression index (IGC)						4.79

### Solubility studies for pH 1.2, 4.5, 6.8 and 7.0

CTZ is very slightly soluble at pH 1.2 and 4.5 (acid medium) and very soluble at pH 6.8 and 7.0 (neutral medium).

### NMR, IR, PXRD and UV-Vis: Identification and characterization CTZ features

**Fig 2:**
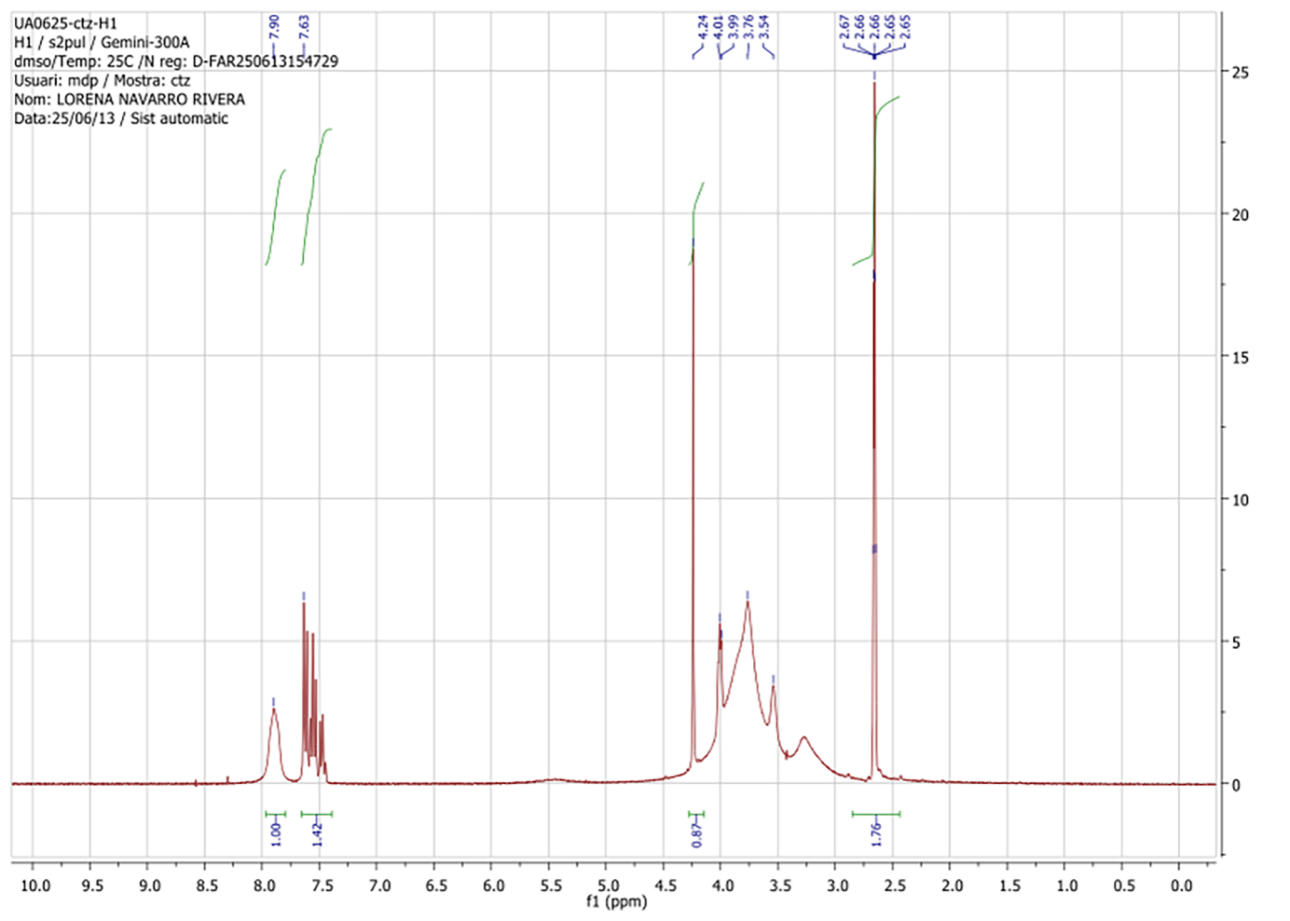
Nuclear magnetic resonance of CTZ.

NMR correctly identified the presence of CH_2_-N from the piperazine ring), the functional group Ar (aryl), the carboxyl group–COOH, and other groups (CH_2_-O) as components of the CTZ (see Fig 2).

**Fig 3:**
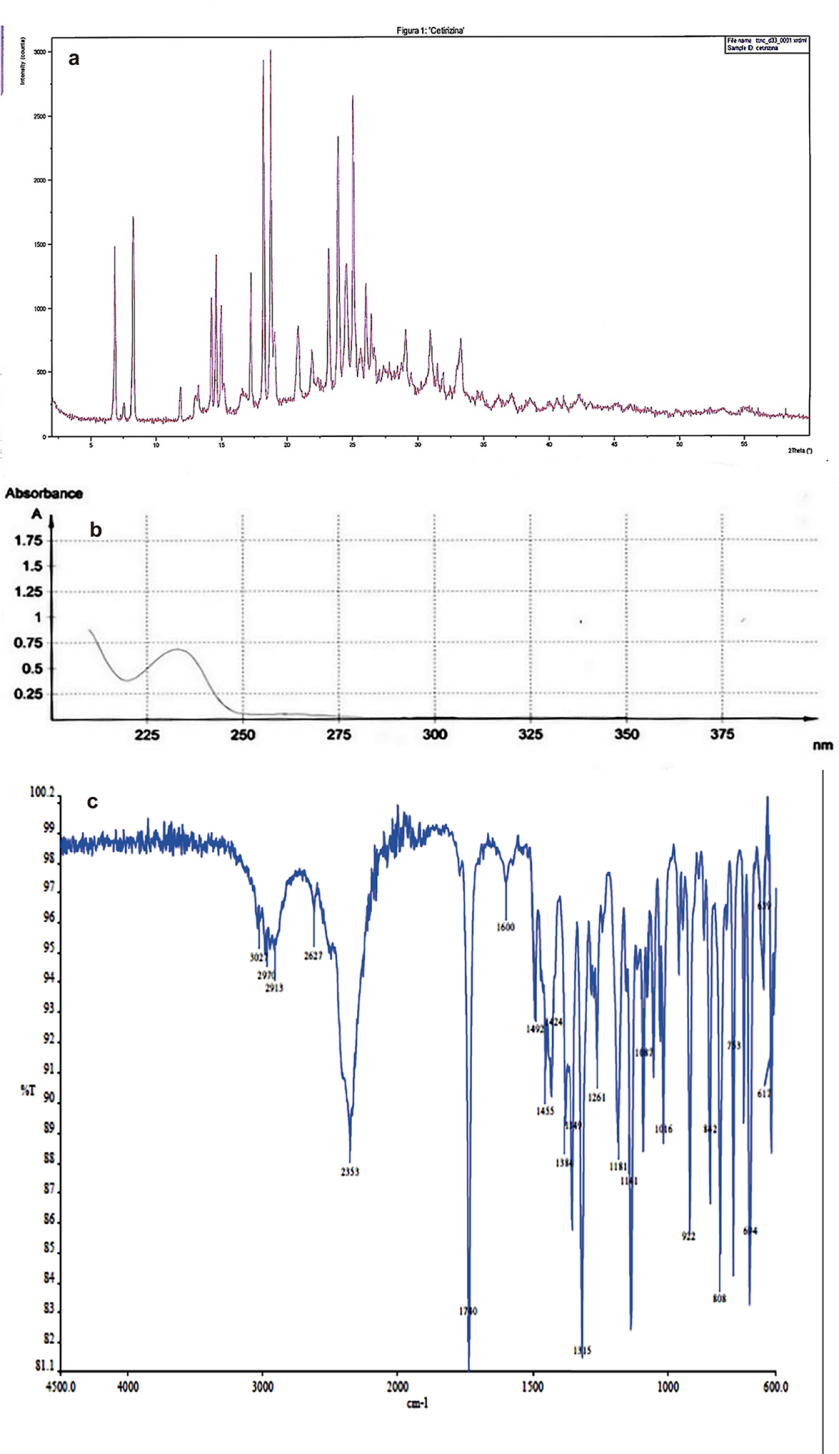
PRDX, UV-Vis and IR of CTZ. Fig 3a PRDX, 3b UV-Vis and 3c IR, respectively.

The IR spectrum showed an absorption peak at 3500 cm^-1^ identifying the hydroxyl group (-OH), which is part of the carboxylic group–COOH. An absorption peak was also determined at 2627 cm^-1^ identifying the presence of–COO^-^. Another absorption peak at 1740 cm^-1^ was determined, characteristic of the carboxyl functional group -C = O. Different absorption peaks were determined about 1500 cm^-1^, indicating–CH groups, which are present at the aliphatic chain of the molecule. An absorption peak at 1315 cm^-1^ was found, corresponding to a -C-N (indicating the presence of a piperazine ring), and about a 1000 cm^-1^ an identification peak of -C-O and at 612 cm^-1^ there is an absorption peak corresponding to a -C-Cl link (see Fig 3).

As for the PRDX results, the diffractogram of CTZ showed several lines of intensity, indicating that CTZ is at a crystalline state. Fig 3 shows the diffraction diagram of CTZ obtained in the main angular range of 2 to 60°2θ.

The UV-Vis spectrophotometry technique confirms the maximum absorption wavelength (λ) at 231 nm, complying with the specification of Ph. Eur. for CTZ (see Fig 3).

## Design of experiments (DoE)

### Freeze-drying viability tests (FDVT)

FDVT were performed to determine the concentration of bulking agents (mannitol and PVP K30) and the matrix consistency obtained with CTZ, as the final filling dose equivalent of 10 mg of active substance.

### In crystal vial

Freeze drying of 1 ml solution of 1% CTZ (W/v) and 0.25, 0.5, 1.0 and 2.0% mannitol (W/v) tested presented cracked plugs, although due to the higher concentration of mannitol, the concentration of 2.0% (W/v) was considered acceptable for further test with PVP K30.

A second batch of 1 ml solution of 1.0% CTZ (W/v) and PVP K 30 at 1.0, 3.0 and 5.0% (W/v), presented all acceptable plugs, with the 5.0% (W/v) concentration plug with a more compact aspect. A third batch was then performed with 1ml of 1.0% CTZ (W/v), 2.0% (W/v) mannitol and 5.0% PVP K30 (W/v). The combination of CTZ, mannitol and PVP K30 presented a final acceptable result, with no apparent cracking, named **X**_**1**_.

### In PVC mold

A fourth batch was performed with **X**_**1**_ and two different PVC molds, maintaining the 1 ml filling dose. The first mold had round alveolus of 5 mm high x 15 mm diameter and the second mold had oval alveolus of 10 mm high x 15 mm long. **X**_**1**_ presented a cracked aspect with the round alveolus, and a better aspect with the oval alveolus. With the aim of reducing the size of the OL, a fifth batch was carried out, maintaining the same concentration of bulking agents and increasing the concentration of CTZ to a 5.0% (W/v) to fill with 0.2 ml of solution (**X**_**2**_) into smaller round alveolus of 4 mm high x 10 mm diameter. **X**_**2**_ presented again a cracked aspect.

A sixth batch was tested, maintaining the same concentration of bulking agents and reducing the concentration of CTZ to a 2.0% (W/v)—solution **X**_**3**_ –with a round alveolus mold, with 0.5 ml volume (with 5 mm high x 12 mm diameter). **X**_**3**_ obtained had no cracked aspect, although presenting a fragile consistency when extracted from the PVC mold (Table 6).

**Table 6:** First FDVT in PVC molds.

X (formula)	ml = 10 mg dose	CTZ % (W/v)	Mannitol % (W/v)	PVP K30% (W/v)	alveolus shape mold	alveolus dimensions mold	Final aspect (cracked/not cracked)
X1	1	1.0	2.0	5.0	Round	5 mm high x 15 mm diameter	Cracked
					Oval	10 mm high x 15 mm long	Not cracked
X2	0.2	5.0	2.0	5.0	Round	4 mm high x 10 mm diameter	Cracked
X3	0.5	2.0	2.0	5.0	Round	5 mm high x 12 mm diameter	Not cracked

Therefore, it was determined the concentration of CTZ for the freeze-dried solution **X**_**3**_: 2% CTZ (W/v), the alveolus for the PVC molds (round alveolus with 5 mm high x 12 mm de diameter) and the filling dose (0.5 ml = 10 mg). Also, a starting combination of bulking agents was established of 2.0% (W/v) mannitol and 5.0% (W/v) PVP K30.

To acknowledge whether this proportion between mannitol, PVP K30 and CTZ could present metastable forms, a factorial design was carried out with different proportions of the bulking agents and CTZ.

### Determination of Tg’ and detection of metastable forms

**Fig 4:**
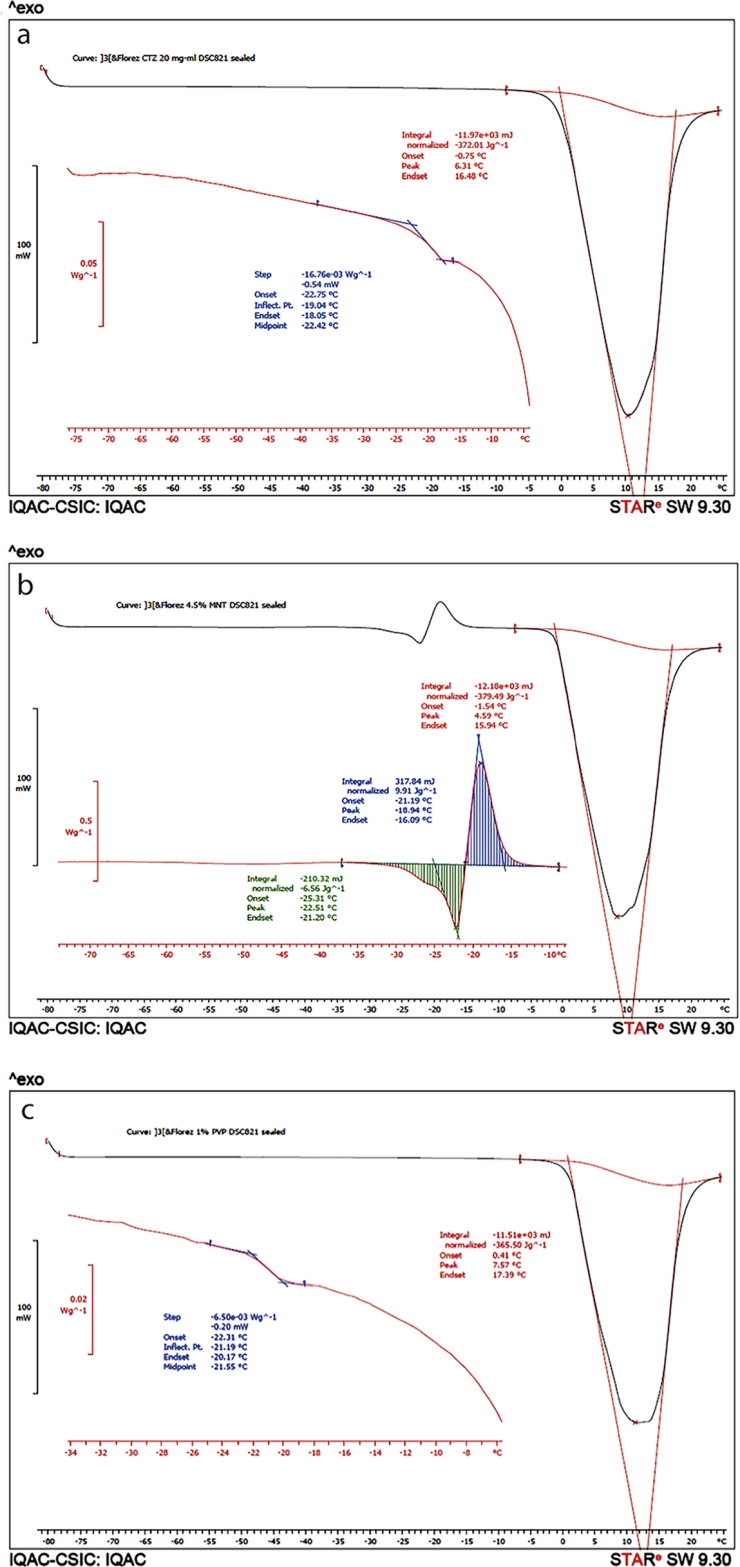
DSC of CTZ, mannitol and PVPK30. Fig 4A and Fig 4C without metastable forms, Fig 4B with metastable forms.

A solution of 2% (W/v) CTZ, 2.0, 4.5 and 7.0% (W/v) mannitol and 1.0, 3.0 and 5.0% (W/v) PVP K30 solutions were analyzed separately (Fig 4). The results showed that CTZ and PVP K30 did not presented metastable forms. On the other hand, all concentrations studied of mannitol presented metastable forms (see Table 7).

**Table 7:** Determination of Tg’ and metastable forms of CTZ and excipients by DSC.

Sample	Metastable forms (presence/absence)	Tg’ (°C)
CTZ 2.0%	Absence	**-22.75**
MNT 2.0%	Presence	**-27.46**
MNT 4.5%	Presence	**-25.31**
MNT 7.0%	Presence	**-25.28**
PVP K30 1.0%	Absence	**-22.30**
PVP K30 3.0%	Absence	**-25.41**
PVP K30 5.0%	Absence	**-33.72**

### 3^2^ Factorial design (effect of mannitol and PVP K30)

A simple factorial design was carried out with two previous conditions considered for determining the best combination of excipients: to choose a solution without metastable forms (in order to ensure drug stability) and with a Tg’ superior to -40°C (which was the maximum freezing temperature applied by the freeze-drying equipment). Once determined the calorimetric feature of each component, the factorial design 3^2^ (see Table 8) was performed, maintaining the concentration of 2.0% CTZ (W/v). Hence, nine solutions named A, B, C, D, E, F, G, H and I were studied (see Table 8).

**Table 8:** 3^2^ Factorial design results of DSC and FDM.

Solution	% Mannitol*	% PVP K30*	Metastable form (Absence/Presence)	Tg’°C (DSC)	T_CO_°C (FDM)	Second lyophilisation viability test (Yes/No)
A	2.0	3.0	Absence	-29.1	-29.0	Yes
B	4.5	3.0	Presence	-36.0	-34.0	No
C	7.0	3.0	Presence	-30.4	-29.0	No
D	2.0	5.0	Absence	-27.3	-31.0	Yes
E	4.5	5.0	Absence	-32.2	-31.0	Yes
F	7.0	5.0	Presence	-33.2	-39.0	No
G	2.0	1.0	Presence	-34.0	-36.0	No
H	4.5	1.0	Presence	- 29.9	-25.0	No
I	7.0	1.0	Presence	-29.5	-35.0	No

*(W/v).

**Fig 5:**
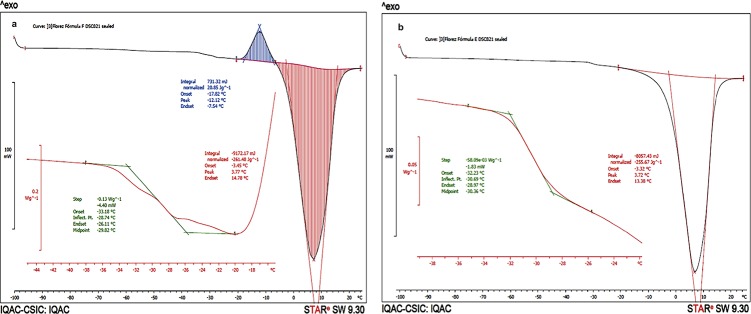
DSC of solutions F and E. Fig 5A shows a DSC of Solution F with metastable forms, and Fig 5B shows a DSC of Solution E, without metastable forms.

All solutions presented Tg’ superior to– 40°C. Solutions A (2.0% mannitol and 3.0% PVP K30), D (2.0% mannitol and 5.0% PVP K30) and E (4.5% mannitol and 5.0% PVP K30) did not present metastable forms, and all three presented a higher proportion of PVP K30 than mannitol. On the contrary, solutions B, C, F, G, H and I (Fig 5) presented higher proportions of mannitol that PVP K30 and metastable forms (Flórez Borges et al., 2015).

### Freeze-drying microscopy (FDM) of 3^2^ factorial design

**Fig 6:**
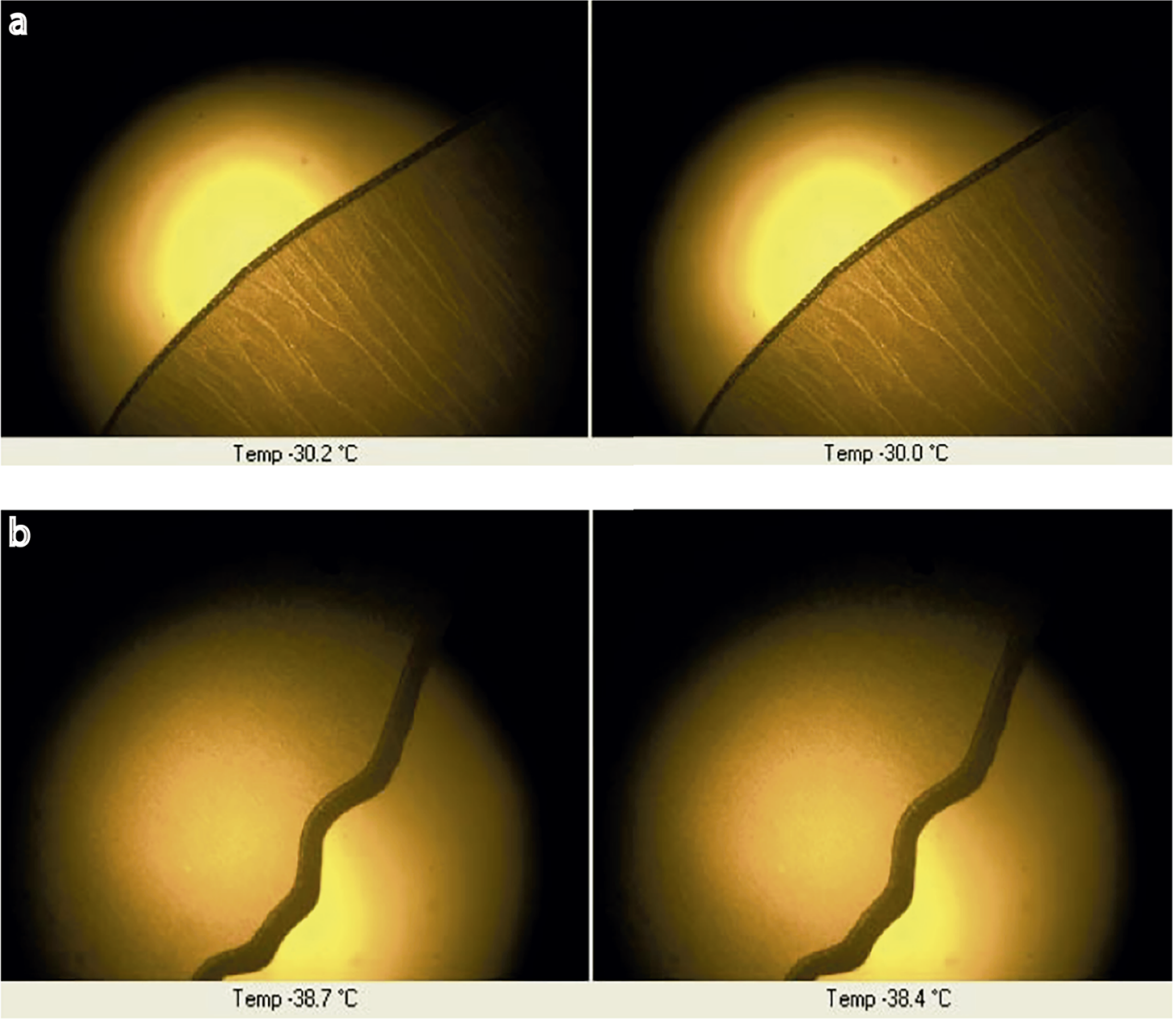
FDM of solutions E and F (a and b, respectively).

The results obtained from the FDM technique were considered as a tool for obtaining the collapse temperature (T_CO_) of each solution of the factorial design. T_CO_ is the temperature where the product loses its structural rigidity and collapses [7]. As for the process of freeze drying, the product must cool for complete solidification below Tg’ and T_CO_, and the FDM technique can be used to identify visibly T_CO_, providing complementary information to ensure that the cycle applied (- 40°C– 40°C) was suitable for the formulation. Observing the results obtained (see Table 8) it was observed that solutions A, D and E (without metastable forms) presented similar T_CO_ (approximately -29.0, -31.0 and -31.0°C, respectively), whereas solutions B, C, F, G, H and I (which presented metastable forms) presented T_CO_ ranging from -25.0°C to -39°C (Fig 6). The freeze-drying cycle applied was considered suitable for formulations A, D and E as it reached -40°C, which guarantees that each formulation is frozen below its critical temperatures, Tg’ and T_CO_.

### Second FDVT

Solutions A (2.0% mannitol and 3.0% PVP K30), D (2.0% mannitol and 5.0% PVP K30) and E (4.5% mannitol and 5.0% PVP K30), with similar results of Tg’ and T_CO_ (approx. = -30.0°C, respectively), and no metastable forms, were tested with the same freeze-drying cycle (-40°C to 40°C) and PVC mold previously established (Table 6, example **X**_**3**_). Solution A matrix presented a white translucent color with a cracked aspect inside the plug and broke easily when extracted from the PVC mold; the same occurred with solution D plug. Both matrix A and D presented the same proportion of mannitol (2.0%) and different proportions of PVP K30 (3.0% and 5.0%, respectively). Solution E plug presented a more compact aspect (white color with no translucency) and less fragility when extracted from the PVC mold due to an increase of solid content with 4.5% mannitol in comparison with solutions A and D.

## Discussion

The aim of this preformulation study was to develop an oral lyophilizate using CTZ as active ingredient model. An important aspect of this study is the predetermined and broad (-40 to 40°c) freeze-drying cycle used, with no possibility of applying any changes, such as annealing, for instance. Therefore, this boundary had set three main objectives: achieving a formula with good consistency, absence of metastable forms and Tg’ and T_CO_ that could fit into the cycle, and not otherwise.

For stability purposes, the use of SeDeM diagram provided valuable data regarding the stability behavior of CTZ in its solid form. For instance, CTZ presented a humidity content of 0.150% and a spontaneous hygroscopicity of 0.200%, a positive trait for the final stability of an OL. Also, by DSC, CTZ presented a good calorimetric profile, with no metastability detected.

In terms of matrix consistency, FDVT were performed observing aspects such as concentration of bulking agents, active ingredient and PVC molds; mannitol and PVPK30, were both initially studied as main bulking agent separately. If only considering the matrix consistency, mannitol would be the only bulking agent chosen due to the fact that in the first FDVT it presented a good plug consistency in vial with 2%. However, according to DSC determination of the different concentrations of mannitol studied (2, 4.5 and 7.0%), it is not recommended to be used as sole excipient, as it presented metastable forms. Therefore, the addition of PVP K30, which from the start presented a good calorimetric profile with absence of metastable forms (Fig 4C and Fig 5B), indicates its value as bulking agent with mannitol, conferring the aimed calorimetric stability needed and adding its valuable characteristics as bulking agent for freeze dried formulas as well. From the simple 3^2^ factorial design, it was observed that, with the crescent addition of PVP K30, the metastable forms disappeared (Table 8). Nevertheless, Tg’ started to increase, which also is an important aspect to be considered while working within a predetermined cycle. To sum up, the SeDeM method was useful in characterizing CTZ and giving information regarding its humidity content and hygroscopicity, as PVP K30 was key to eliminate the metastable forms generated by mannitol. Finally, solution E (4.5% mannitol and 5% PVPK30) was considered the best combination in order to obtain the final OL matrix for CTZ, by presenting a Tg’ of 32.2°C, T_CO_ of 31°C, absence of metastable forms, a less brittle and more compact aspect when extracted from the round PVC mold of 5 mm high x 12 mm diameter.

## Abbreviations

CTZ: cetirizine dihydrochloride
DSC: Differential scanning calorimetry
PVP: polyvinylpyrrolidone
FDM: freeze drying microscopy
Tg’: glass transition temperature
T_CO_: collapse temperature
NMR: nuclear magnetic resonance
IR: infrared spectroscopy
UV-Vis: ultraviolet-visible
PRXD: powder method of X-ray diffraction
OL: oral lyophilizates
DoE: design of experiments
FDVT: Freeze-drying viability studies
%HR: loss on drying
IcD: Cohesion index
IP: parameter index
IPP: parameter profile index
ICG: good compression index
Dc: tapped density
Da: bulk density
Ie: inter-particle porosity
PVC: polyvinyl chloride
